# Messenger effects in COVID-19 communication: Does the level of government matter?

**DOI:** 10.1016/j.hpopen.2020.100027

**Published:** 2021-01-08

**Authors:** Nathen Favero, Sebastian Jilke, Julia A. Wolfson, Chengxin Xu, Matthew M. Young

**Affiliations:** a American University, School of Public Affairs, Kerwin Hall – 340, Washington, DC 20016, United States; b Georgetown University, McCourt School of Public Policy, 37th & NW O Streets, Old North – 100, Washington, DC 20057, United States; c University of Michigan, School of Public Health, 1415 Washington Heights, Ann Arbor, MI 48109, United States; d Institute of Public Service, Seattle University, 901 12th Avenue, Casey 210-09, Seattle, WA 98122, United States; e Syracuse University, Maxwell School of Citizenship and International Affairs, 200 Eggers Hall, Syracuse, NY 13244, United States

**Keywords:** COVID-19, Messenger effect, Health messaging, Survey experiment

## Abstract

Public efforts to limit the spread of the coronavirus rely on motivating people to cooperate with the government. We test the effectiveness of different governmental messengers to encourage preventive health actions. We administered a survey experiment among a sample (n = 1,545) of respondents across the United States, presenting them with the same social media message, but experimentally varying the government sender (i.e., Federal, State, County, a combination of Federal + County, and a control condition) to test whether local relevance influences messaging efficacy. We find that in an information saturated environment the messenger does not matter. There is, however, variation in treatment response by partisanship, education, income, and the degree to which respondents are affected by the pandemic. While the main effect of the level of government on intended behavior is null, public health organizations are universally perceived as more trustworthy, relevant, and competent than anonymous messengers.

## Highlights

• We conducted a large-scale survey experiment (n = 1,545) early in the COVID-19 pandemic.
• The sender of a hypothetical public health message was randomly assigned.
• As outcome we measure people’s motivation to engage in preventive health actions.
• No statistically significant differences between senders (different government levels versus no sender.
• Heterogenous effects in terms respondents’ partisanship, education, income and pandemic exposure.
• COVID-19 public health communication needs to be targeted toward specific population segments.

## 1 Introduction

The rapid spread of COVID-19 in the United States (U.S.) has necessitated the adoption of mitigation efforts involving changes to daily life that have upended society as we know it. During the course of the pandemic, government agencies have regularly asked people across the United States to stay at home and closely follow public health guidance on social-distancing, self-isolation and hygiene practices. As of April 20, people in at least 42 states, the District of Columbia and Puerto Rico had been urged by the government to stay home [1]. However, even when governments have issued mandatory stay-at-home orders in the U.S., there remains substantial variation in individual compliance -- mobile phone data shows that only about 40% of the nation stayed at home from late March through mid April 2020 [2]. A large-scale convenience survey conducted between March 14–23 found that about 40% of respondents reported not being compliant with self-isolation orders [3]. Many states subsequently relaxed restrictions on businesses and personal gatherings (as of July 9, 13 states have reopened, and 17 states and the District of Columbia are reopening in stages [4]), thus leaving more decisions about responsible behavior up to individuals. In addition to mandating business closures or masks in public, government also issues messages about practices and behaviors aimed at limiting the spread of COVID-19. Especially as the political will for mandated restrictions wanes, efforts to limit the spread of COVID-19 necessarily rely on motivating the public to cooperate with public health recommendations [5].

Government agencies use various messaging strategies to increase public health policy compliance among the general public. Public health messages have been found to be effective in encouraging preventative actions across various domains, such as encouraging tests for HIV [6] and Ebola vaccination [7]. During the COVID-19 pandemic, research has examined how message content increases compliance with public health messaging. But there is substantial variation in messaging effectiveness. Some studies have highlighted the importance of empathy and pro-socially framed messages [8], [9]. Others did not detect substantial differences in people’s willingness to comply due to differences in message content [10], [11], [12].

While public health agencies and other government entities employ different messaging strategies to varying degrees, little attention has been paid to the level of government encouraging citizens to engage in preventive health practices via public messaging. This is surprising, because the public is strongly influenced by the messenger of information. Indeed, messenger effects (often also referred to as source or communicator effects) have been shown to strongly affect behavior change [13], [14]. Messenger effects are typically studied by comparing lay citizens to experts; for instance a high-school teacher versus a public health official [11], a celebrity versus a government official [15], or a neighbour versus the government [16]. In this study, we test for COVID-19 related messenger effects with regard to the level of government using an online survey experiment in which we created a hypothetical social media post emphasizing COVID-19 infection severity and encouraging the public to help slow its spread through hand washing and social distancing. We experimentally varied the source of the post while holding the message content constant.

## 2 Study data and methods

*Data:* We created a web-based survey experiment on the Qualtrics platform. The survey was fielded between March 27 and April 10, 2020. The survey was pre-registered, and quota-sampling was used to ensure the study sample was representative of the US population based on age, gender, race/ethnicity, and region (Northeast, South, Midwest and West). Respondents were further screened by Qualtrics for response quality; this was conducted independent of the research team. The final sample (after Qualtrics quality screening independent of the research team) was 1,545 adults (18 years or older) in the US. *A priori* calculations indicated that a sample size of 1,500 would allow a minimum detectable effect between treatment conditions of 3.7 percentage points at 80% power.

Respondents were presented with a hypothetical social media post emphasizing the severity of COVID-19 infections and encouraging them to help slow its spread through hand washing and social distancing. The hypothetical post read “The number of confirmed COVID-19 cases in the U.S. increased 337% from March 20 to 25 (19,582 to 65,982 cases). Please follow the guidelines below to help slow the spread: avoid crowds, wash your hands, stay home, if you feel sick, self-isolate.” We experimentally varied the source of the post while holding its content constant. Respondents were randomly assigned to one of five experimental conditions:

1. Control (no identified source);
2. Federal (Center for Disease Control (CDC) as the identified source);
3. State (respondent’s state department of health as the identified source);
4. County (respondent’s county department of health as the identified source); or
5. A combination of county and federal (respondent’s county department of health as the identified source of a message produced in association with the CDC).

For treatments (3) through (5), the respondent’s actual state or county was named as the source of the hypothetical post (based on a ZIP code supplied by the respondent earlier in the survey). Conditions (2) through (4) gradually increased the local relevance of the message while holding expertise-based competence constant because all messengers are identified as departments of public health. The fifth condition combines treatments (2) and (4), allowing us to verify that perceived messenger competence does not affect differences between (2) through (4). Full treatment materials can be found in the Appendix.

*Outcome measures:* After reading the hypothetical social media post and answering a brief manipulation check, respondents answered a set of questions indicating first-order (reporting one’s own likely response) and second-order (perceptions of others’ likely responses) behavior regarding hand washing, social distancing (staying at home and avoiding social contact), and likelihood of sharing the post. Specifically, respondents were asked how likely they/others were to “wash your hands whenever you enter work or come home for at least 2 weeks, even if you don’t feel sick?”; how likely they/others were to “stay at home and completely avoid all social contact for at least 2 weeks, even if you don’t feel sick?”; and how likely they/others were to “share this post to your own social media.” All outcomes were measured on a 0–100 point slider with 0 = ”extremely unlikely” to 100 = ”extremely likely.” The order of questions was randomized to avoid priming effects.

A set of secondary outcomes consists of responses to questions soliciting perceptions of the messenger’s competence, relevance, and trustworthiness. Specifically respondents were asked to think about the organization that posted the message and then indicate on a 100-point slider: (1) how competent the organization is (0=”extremely incompetent” to 100=”extremely competent”); (2) how relevant the content is for the respondent (0=”not at all relevant” to 100=”extremely relevant”); and, (3) how trustworthy they think the organization is (0=”extremely untrustworthy” to 100=”extremely trustworthy”).

*Other measures:* Socio-demographic characteristics of the study sample included gender (male, female, both/neither/fluid/other), race/ethnicity (non-Hispanic white, non-Hispanic Black, Hispanic, Asian/Pacific Islander, other), age (18–34 years, 35–64 years, 65 years), presence of children in the household, education (high school, some college, college graduate), household income, ($0-$29,999, $30,000-$59,999, $60,000-$99,999, $100,000 or more), employment status (employed, unemployed, retired/student/disabled), political party affiliation (Democratic, Republican, other), and presence of underlying health conditions (yes, no, unsure).

We included controls for state-level COVID-19 cases per capita (100 k), and state level COVID-19 deaths per capita (100 k), as of the date each respondent took the survey. As an additional control we measured how closely the respondent had followed COVID-19 news using a 4-point Likert scale from “not at all closely” to “very closely”, and fear of exposure to COVID-19 measured on a 0–100 slider. Finally, we also include a measure of respondents’ county urbanity (rural, urban) from the 2010 Decennial Census. *Statistical Analysis:* First, we used cross-tabulations and chi-squared statistics to describe the study sample and test for differences across study arms. We used violin plots to display the distribution of all study outcomes.

We then estimated the effect of study arm assignment on primary and secondary outcomes using a tobit model with right-censoring. The tobit specification was chosen because many respondents selected maximal values (1 0 0) for their outcome measure responses. Estimated coefficients are interpreted the same way as OLS coefficients. The key independent variable was an indicator of the study arm to which the participant was randomized. We estimated unadjusted treatment effects as well as models adjusted for respondent characteristics and the respondents’ state per capita COVID-19 cases and deaths at the time they took the survey. In addition, we estimated several sensitivity analyses, including testing for heterogeneous treatment effects by interacting respondent characteristics with treatment assignment. Post estimation margins commands were used to show predicted outcomes based on interaction models.

Analyses were performed using STATA Version 15 in 2020, all tests were two sided and significance was considered at *p* < 0.05. All reported significance levels were validated under multiple hypothesis test conditions using the Benjamini-Hochberg procedure [17]. We use a significantly restrictive false discovery rate threshold (0.05) for testing the main treatment effects. We use a lower threshold (0.25) for testing heterogeneous treatment effects because these are exploratory analyses where the risk of Type II errors needlessly precluding future research is of greater concern [18].

*Limitations*: There are several limitations to bear in mind when interpreting our results. While our sampling frame was designed to be nationally representative, web-based instruments may limit representation among groups that do not have internet access or are less internet-savvy. Survey participants were asked to self-report their responses to a hypothetical social media post. While attempts were made to make the hypothetical post feel realistic by showing respondents an image that mimics the style of a real social media post, it is possible that people would respond differently to such posts coming from a real poster on social media. Self-reporting of behavior can diverge from actual behavior, especially when there are clear socially desirable answers, although prior research suggests that self-reported measures of social distancing behaviors track fairly well with other measures of activity that do not rely on self-reports [19].

When it comes to generalizing from our results, it is important to bear in mind the timing of our study. The survey was fielded in late March through early April, a period during which there appeared to be a somewhat fragile consensus across political parties on the need to adopt strong mitigation measures, as evidenced by the adoption of stay-at-home orders by most state governments throughout the U.S. Gallup polling indicates the gradual emergence since then of a substantial partisan gap in self-reported social distancing behaviors and attitudes [20]. Given stark partisan differences, the public may now interpret messages through a more partisan lens, perhaps responding more positively to messengers who they believe are aligned with their own political perspective. A surge in newly confirmed cases seen in late June in several states throughout the U.S., particularly in the South and West, has brought renewed calls for social distancing and mask wearing from some prominent Republican officials across different levels of government [21], [22].

## 3 Study results

Table 1 presents descriptive statistics for all respondents, compares those against national averages from 1-year 2018 American Community Survey estimates. The study sample closely mirrors the national population on gender, age, race/ethnicity, educational attainment, and region of the country. Randomization across study arms was successful.

**Table 1:** Descriptive statistics of respondent characteristics.

	Number	Percent	National Comparison	Assignment Arm Randomization
**Gender**				
Female	787	51%	51%	Pearson X^2^ = 2.12 p = 0.71
Male	749	48%	49%	
Neither/Fluid/Other	9	1%	–	
**Age Cohort**				
18–24	194	13%	12%	Pearson X^2^ = 32.07 p = 0.27
25–34	274	18%	18%	
35–44	258	17%	16%	
45–54	275	18%	16%	
55–64	253	16%	17%	
65 or older	291	19%	21%	
**Race and Ethnicity**				
White	959	62%	60%	Pearson X^2^ = 5.97 p = 0.20
Black or African American	189	12%	12%	
Hispanic, Latino, Spanish	269	17%	18%	
Asian and Pacific Islander	91	6%	6%	
Other	37	2%	4%	
**Educational Attainment**				
Less than high school	199	13%	12%	Pearson X^2^ = 35.22 p = 0.065
High school graduate	429	28%	27%	
Some college	448	29%	29%	
4 year degree	292	19%	20%	
Graduate degree	150	10%	11%	
Doctorate	27	2%	2%	
**Employment Status**				
Employed	769	50%	60%	Pearson X^2^ = 14.12 p = 0.08
Unemployed	279	18%	3%	
Retired/Student/Disabled	497	32%	37%	
**Household Income** **by Quartile**				
1st (0-$29,999)	674	44%	24%	Pearson X^2^ = 15.69 p = 0.21
2nd ($30,000-$59,999)	428	28%	25%	
3rd ($60,000-$99,999)	267	17%	23%	
4th ($100,000 + )	176	11%	29%	
**Partisanship**				
Democratic	692	45%		Pearson X^2^ = 7.97 p = 0.44
Republican	528	34%		
Other	325	21%		
**Region**				
Northeast	308	20%	17%	Pearson X^2^ = 16.72 p = 0.16
South	575	37%	38%	
Midwest	310	20%	21%	
West	352	23%	24%	

Source: Authors’ own analysis. Notes: Socio-demographic comparison data drawn from 2018 American Community Survey 1-year estimates.

**Fig. 1:**
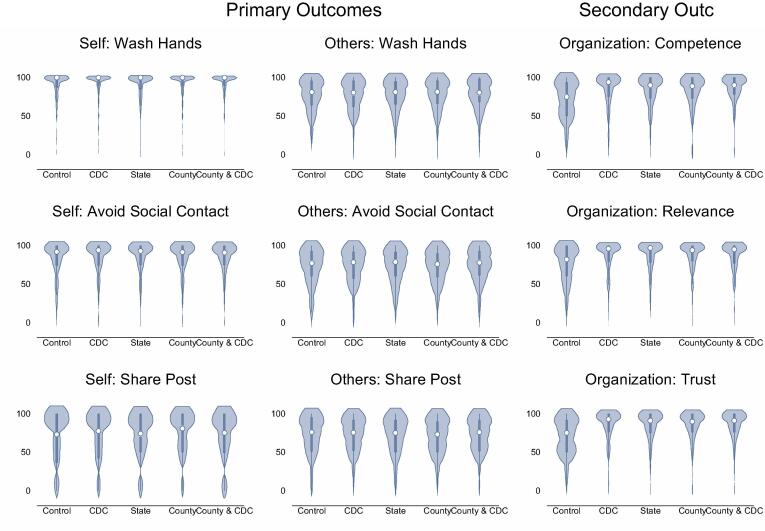
Violin Plots of Unconditional Primary and Secondary Outcomes. Source: Authors’ own analysis.

Fig. 1 shows the distribution of primary and secondary study outcomes across study arms using violin plots. Across study arms, respondents showed clear differences in the effect they thought the social media message would have for others compared to for themselves. There are also clear, systematic differences in the secondary assessment measures of competence, relevance, and trustworthiness between the control and treatment arms.

We consistently find null effects for our primary outcomes (Table 2). None of our treatments yield a statistically significant difference from the control group, which was shown a post from an unknown messenger. There are also no statistically significant differences among the various experimental conditions. The lack of statistical significance is not driven by large standard errors; all slope coefficients have standard errors in the range of 2–4 points (compared to the 100-point scale for our outcomes). Instead, insignificance is driven by the modest magnitude of our coefficient estimates, which generally have an absolute value of 3 or smaller (the largest is 5.5). Results remain statistically insignificant, despite slightly smaller standard errors, when covariate adjusted for pre-treatment variables, including demographic characteristics, political party preference, and news attentiveness, concern, and potential vulnerability regarding COVID-19. Our null findings are also robust to a complier average causal effect (CACE) specification.

**Table 2:** Regression estimates of primary and secondary outcomes.

	Primary Outcomes						Secondary Outcomes		
	Self			Other			Organization		
	Wash	Avoid	Share	Wash	Avoid	Share	Competence	Relevance	Trust
CDC	3.85	2.49	0.76	−0.68	0.38	−1.06	16.83^***^	16.26^***^	18.88^***^
	(2.49)	(2.54)	(3.38)	(2.08)	(2.07)	(2.36)	(2.33)	(2.45)	(2.32)
State	1.61	1.16	−1.42	−0.37	0.31	−2.83	14.61^***^	16.01^***^	19.25^***^
	(2.46)	(2.52)	(3.36)	(2.07)	(2.06)	(2.34)	(2.31)	(2.44)	(2.31)
County	2.88	0.78	2.58	−1.23	−1.44	−2.64	11.14^***^	14.17^***^	17.12^***^
	(2.48)	(2.54)	(3.38)	(2.08)	(2.06)	(2.35)	(2.3)	(2.43)	(2.30)
County & CDC	4.08	1.81	−0.48	1.74	1.51	−1.32	15.90^***^	15.45^***^	19.20^***^
	(2.49)	(2.52)	(3.37)	(2.09)	(2.07)	(2.35)	(2.32)	(2.43)	(2.33)
Observations	1,545	1,545	1,545	1,545	1,545	1,545	1,545	1,545	1,545

Source: Authors’ own analysis. Notes: Control assignment used as reference category. Standard errors in parentheses. * p < 0.05 ** p < 0.01 *** p < 0.001 Significance values are robust to a False Discovery Rate of 0.01 using the Benjamini-Hochberg procedure. Estimated using a tobit specification with right-censoring at 100.

For our secondary outcomes, all 4 treatments have statistically significant and substantively meaningful effects relative to the control (Table 2). Respondents indicate improved assessments of the poster’s competence, relevance, and trustworthiness when it is a government organization as opposed to an unknown entity. At the same time, no statistically significant differences among the various treatment groups were detected. It does not matter *which* government organization acted as the messenger, just that *a* government organization was the messenger. The named sources receive an estimated 11–19 point bump on the 100 point scales relative to the unnamed poster. These results are robust when we adjust for covariates or estimate CACE (see Appendix).

**Fig. 2:**
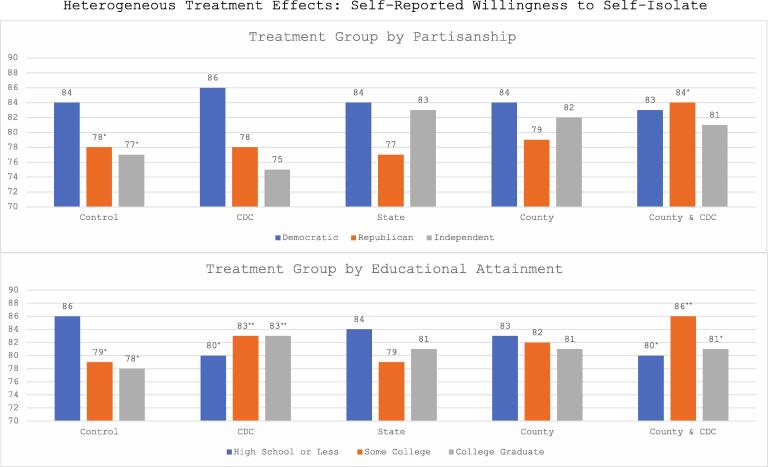
Heterogenous Treatment effects of partisanship and educational attainment on self-reported willingness to self-isolate. Source: Authors’ own analysis. Notes: * p < 0.05 ** p < 0.01 *** p < 0.001.

Finally, we tested for heterogeneous treatment effects by several demographic and geographic covariates [23]. Self-reported intention to engage in social distancing is moderated by both partisanship [24] and education (Fig. 2). Republican and independent respondents reported lower values than democrats irrespective of treatment assignment. However, this effect was reversed on net among those who received the hybrid CDC & County treatment for republicans, who had a higher average reported willingness to socially isolate than either independents or democrats (though this effect is marginal at p = 0.052). For education, those with some college and those with college degrees report lower willingness to socially isolate than those with a high school diploma or less irrespective of treatment assignment. But both some college and college graduate groups respond positively to CDC-only and CDC & County treatment; exposure to these treatments left both groups more willing to socially isolate than those with a high school education or less.

Additional tests for heterogeneous treatment effects are shown in the Appendix. No significant differences in treatment effects were found by gender. There are no consistent patterns of heterogeneous reactions to treatments by age or employment status, although for each of these characteristics, one to three interaction coefficients attain statistical significance.

## 4 Discussion

In the context of the early days of the COVID-19 pandemic in the U.S., we do not detect governmental messenger effects with regard to people’s intentions to engage in preventive health measures, as well as their perceptions that other people would do so. Consistent with a theory of information overload with regard to COVID-19 messaging [7], it is likely that respondents who would have been receptive to our intervention had already seen actual government messages prior to taking our survey and adjusted their behaviors accordingly. Many states were also announcing formal stay-at-home orders during the time of our study, with 42 states having issued such orders by April 20, 2020 [1]. This may explain the relatively high levels of compliance with public health recommendations and positive attitudes among our sample at the beginning of the study. We also note that high compliance rates per se can produce ceiling effects as visualized in Fig. 1. When compliance rates are close to the ceiling, the effect of additional interventions, including messaging campaigns, is correspondingly smaller, which in itself may be a driver of our results. However, differential results based on partisanship and education demonstrate that compliance was not universal, and opportunities exist for targeted public health messaging tailored to different demographic groups.

Interestingly, though we did find differences in competence, relevance and trust of the various government messengers relative to the control group, we were not able to detect statistically significant differences between messengers from different levels of government with regard to perceptions of the messenger’s competence, relevance and trust. If anything, the CDC is perceived as the institution with the highest relevance and competence (albeit differences are not statistically significant), which cuts against the argument that local sources are the most effective messengers [25], [26]. Specifically, for all three measures, the combined CDC & County group improved people’s subsequent ratings relative to the County only condition somewhat, though not enough to attain statistical significance. To the extent that CDC messaging may be perceived as most credible, this could be a reflection of its positive agency reputation [27], [28].

Relative to the control of an unknown poster, the CDC message does sometimes appear more likely to spur behavioral intentions and positive attitudes, especially for those who live in areas with higher levels of infections and deaths, and those with higher educational attainment and income.

In sum, this is evidence against our initial predictions of a messenger effect favoring local government actors. Indeed, which level of government sends a message seems not to be of much importance in an information saturated environment. However, whether these results hold as time goes on, quarantine fatigue sets in [29], government restrictions ease, and people start going back outside regularly is an empirical question and should be subject to further empirical investigation.

## 5 Policy implications

Our findings suggest that, early in the trajectory of the COVID-19 pandemic, the public did not especially value receiving information about COVID-19 from more localized officials. This may suggest that state and local officials can largely rely on amplifying federal messaging from the CDC rather than investing significant time in creating their own, localized messaging. Caution is warranted, however, in adopting this approach as the public may at times perceive that local conditions may differ from national trends enough that general federal guidance is not sufficiently relevant to their own situation. While we found no evidence that such reasoning pervaded our respondents’ thinking, there was greater uniformity in state policy at the time of our study than has been apparent more recently as states have begun reopening in a staggered fashion. To the extent that local officials do detect scepticism among local residents regarding the applicability of federally-derived guidelines, local officials should be ready to step in and communicate to the public their own assessments of the local situation and how federal guidelines might be best applied locally.

The heterogeneous effects we document especially with regard to political affiliation and education also underscore the importance of targeted messaging, and intentional choices of messenger for reaching different demographic groups. To the extent that partisanship now appears to underlie substantial differences in behavior and attitudes regarding mitigation efforts [20], it may be especially beneficial for the public to hear from co-partisan political leaders who affirm the importance of adhering to public health guidelines.

Because our survey was launched prior to the CDC beginning to recommend that people who are not feeling sick should wear cloth face coverings in public, we did not include questions about wearing cloth face coverings. However, as the wearing of face coverings is now considered an important part of national mitigation efforts [30], guidance on face coverings now constitutes a key element of public messaging on COVID-19 for health officials. Face coverings have also become a highly politicized recommendation (much more so than the guidance included in our hypothetical social media post). Therefore, future research to test effective public health messages and messengers to encourage face coverings is warranted.

## 6 Conclusion

Conducted among a large, quota-sampled sample of U.S. respondents, this study found no evidence for the expectation that the level of government matters for COVID-19 related health messaging early on in the course of the COVID-19 pandemic. The messenger of a hypothetical social media post did not affect people’s stated first- and second-order public health behaviors. We attribute these findings to the saturated public health information environment in which the study was conducted. It is reasonable to assume -- but remains an open empirical question -- that these effects will not replicate *at other points* in a pandemic, or similar public health crisis, when such information is still regarded as new and people pay great attention to it or when public health recommendations have become politicized and a polarized public is weary of adhering to virus mitigation strategies. Future research should examine messenger effects for public health recommendations especially as government policies and recommendations to address the COVID-19 pandemic have become increasingly politicized.

## CRediT authorship contribution statement

**Nathen Favero:** Conceptualization, Investigation, Methodology, Writing - original draft. **Sebastian Jilke:** Conceptualization, Investigation, Methodology, Writing - original draft. **Julia A. Wolfson:** Writing - review & editing. **Chengxin Xu:** Conceptualization, Investigation, Methodology, Writing - original draft, Conceptualization, Investigation, Methodology, Writing - original draft. **Matthew M. Young:** Conceptualization, Investigation, Methodology, Writing - original draft, Data curation, Formal analysis.

## Declaration of Competing Interest

The authors declare that they have no known competing financial interests or personal relationships that could have appeared to influence the work reported in this paper.
